# Piperacillin/tazobactam-resistant, cephalosporin-susceptible *Escherichia coli* bloodstream infections are driven by multiple acquisition of resistance across diverse sequence types

**DOI:** 10.1099/mgen.0.000789

**Published:** 2022-04-11

**Authors:** Thomas Edwards, Eva Heinz, Jon van Aartsen, Alex Howard, Paul Roberts, Caroline Corless, Alice J. Fraser, Christopher T. Williams, Issra Bulgasim, Luis E. Cuevas, Christopher M. Parry, Adam P. Roberts, Emily R. Adams, Jenifer Mason, Alasdair T. M. Hubbard

**Affiliations:** ^1^​ Department of Tropical Disease Biology, Liverpool School of Tropical Medicine, Pembroke Place, Liverpool, L3 5QA, UK; ^2^​ Centre for Drug and Diagnostics, Liverpool School of Tropical Medicine, Pembroke Place, Liverpool, L3 5QA, UK; ^3^​ Department of Vector Biology, Liverpool School of Tropical Medicine, Pembroke Place, Liverpool, L3 5QA, UK; ^4^​ Department of Clinical Sciences, Liverpool School of Tropical Medicine, Pembroke Place, Liverpool, L3 5QA, UK; ^5^​ Wellcome Sanger Institute, Wellcome Genome Campus, Hinxton, CB10 1SA, UK; ^6^​ Liverpool University Hospital Foundation Trust, Prescot street, Liverpool, L7 8XP, UK; ^7^​ Faculty of Science and Engineering, University of Wolverhampton, Wolverhampton WV1 1LY, UK; ^8^​ Alder Hey Children’s NHS Foundation Trust, Liverpool, L12 2AP, UK; ^9^​ Department of Biosciences, School of Science and Technology, Nottingham Trent University, Nottingham, NG11 8NS, UK

**Keywords:** piperacillin/tazobactam, resistance, AMR, *Escherichia coli*, bloodstream infection

## Abstract

Resistance to piperacillin/tazobactam (TZP) in *Escherichia coli* has predominantly been associated with mechanisms that confer resistance to third-generation cephalosporins. Recent reports have identified *E. coli* strains with phenotypic resistance to piperacillin/tazobactam but susceptibility to third-generation cephalosporins (TZP-R/3GC-S). In this study we sought to determine the genetic diversity of this phenotype in *E. coli* (*n*=58) isolated between 2014–2017 at a single tertiary hospital in Liverpool, UK, as well as the associated resistance mechanisms. We compare our findings to a UK-wide collection of invasive *E. coli* isolates (*n*=1509) with publicly available phenotypic and genotypic data. These data sets included the TZP-R/3GC-S phenotype (*n*=68), and piperacillin/tazobactam and third-generation cephalosporin-susceptible (TZP-S/3GC-S, *n*=1271) phenotypes. The TZP-R/3GC-S phenotype was displayed in a broad range of sequence types, which was mirrored in the same phenotype from the UK-wide collection, and the overall diversity of invasive *E. coli* isolates. The TZP-R/3GC-S isolates contained a diverse range of plasmids, indicating multiple acquisition events of TZP resistance mechanisms rather than clonal expansion of a particular plasmid or sequence type. The putative resistance mechanisms were equally diverse, including hyperproduction of TEM-1, either via strong promoters or gene amplification, carriage of inhibitor-resistant β-lactamases, and an S133G *bla*_CTX-M-15_ mutation detected for the first time in clinical isolates. Several of these mechanisms were present at a lower abundance in the TZP-S/3GC-S isolates from the UK-wide collection, but without the associated phenotypic resistance to TZP. Eleven (19%) of the isolates had no putative mechanism identified from the genomic data. Our findings highlight the complexity of this cryptic phenotype and the need for continued phenotypic monitoring, as well as further investigation to improve detection and prediction of the TZP-R/3GC-S phenotype from genomic data.

## Data Summary

**Impact Statement**

Piperacillin/tazobactam (TZP) is an important first-line antibiotic used for treating serious bacterial infections such as sepsis. The pairing of a penicillin antibiotic with tazobactam, an inhibitor of class A β-lactamase enzymes, blocks the major mechanism of penicillin resistance, providing wide organism coverage. Most isolates of *Escherichia coli* resistant to TZP are also resistant to third generation cephalosporins (3GC), however a phenotype of *E. coli* resistant to TZP but susceptible to 3GC (TZP-R/3GC-S) has recently emerged, and the diversity of resistant mechanisms and population structure of this phenotype remains unknown. In this study, we show that this phenotype is present in diverse sequence types, and found a variety of predicted resistance mechanisms including the hyperproduction of class A β-lactamase enzymes via copy number increases or strong promoters, carriage of inhibitor-resistant β-lactamases, and an S133G *bla*_CTX-M-15_ mutation known to cause TZP resistance. This data shows that the phenotype has emerged in a wide variety of *E. coli* sequence types rather than a result of clonal expansion or horizontal gene-transfer-dependant dissemination. This study represents a significant improvement to our understanding of the TZP-R/3GC-S phenotype, and associated genotypes, in *E. coli *.

Raw read data and assemblies are available at the European Nucleotide Archive (ENA; www.ebi.ac.uk/ena) under BioProject ID PRJNA644114. Previously published sequencing reads used to construct wider phylogenies are also available at ENA under Bioproject PRJEB4681. Detailed per-strain information on accession numbers, resistance profiles, resistance gene predictions and sequence types (STs) are given in Table S2 (available in the online version of this article).

## Introduction

*Escherichia coli* is the most common cause of bacterial bloodstream infections globally [1], accounting for 27 % of all bacteraemic episodes, with a case fatality rate of 12 % [2], and causing 78.8 bloodstream infections per 100 000 people in the UK in 2014 [3]. Antimicrobial resistance (AMR) in *E. coli* is increasingly prevalent [4–6] and extended spectrum β-lactamase (ESBL) production, mediating resistance to third-generation cephalosporins (3GCs) and other β-lactam antibiotics [7], is of particular concern. ESBLs were recorded in approximately 11 % of *E. coli* isolated from bloodstream infections in the UK in 2018 [8].

One strategy to provide therapeutic options for antimicrobial-resistant infections has been the combined use of β-lactamase inhibitors with β-lactam antibiotics to block the activity of β-lactamase enzymes, rendering the bacteria de facto susceptible [9]. The inhibitor tazobactam, which inhibits class A β-lactamases and includes most ESBL enzymes, is commonly utilized in combination with the penicillin class antibiotic piperacillin [10]. Tazobactam is a ‘suicide inhibitor’, as it irreversibly binds to β-lactamases, inactivating the enzyme [11]. Piperacillin/tazobactam (TZP) has broad spectrum activity against Gram-negative and -positive bacteria [12], is well tolerated [13], available for paediatric use, and utilized in the UK as a first-line empirical agent for serious infections, including pneumonia and intra-abdominal infections [14]. Its broad spectrum makes it an important agent for reducing the usage of carbapenem drugs, which are globally important last-line antibiotics. Limiting carbapenem use is a critical element of antimicrobial stewardship and essential for preventing the spread of resistance [15]. Treatment options for carbapenem-resistant bacteria are often limited to poorly tolerated drugs (e.g. colistin or tigecycline) [16]. Whilst TZP does possess *in vitro* activity against ESBLs, the MERINO trial did not demonstrate non-inferiority of TZP to meropenem in treating patients with ESBL *E. coli* and *K. pneumoniae* bloodstream infections [17]. Carbapenems are therefore now recommended for this patient group [18].

In 2018, resistance to TZP occurred in 9.1 % of invasive *E. coli* isolates in the UK [3]. This can be caused by the production of carbapenemase enzymes [19], multiple β-lactamases [20] or ESBLs in combination with increased efflux or porin loss [21], which can also provide resistance to 3GCs. Recently, a phenotype of resistance to TZP with susceptibility to 3GCs (TZP-R/3GC-S) emerged in *E. coli* and *Klebsiella pneumoniae *, indicating the possibility of alternative resistance mechanisms. The major cause of this phenotype is the hyperproduction of class A or D β-lactamases such as TEM-1 [22, 23]. Increased production of β-lactamase overcomes the inhibitive effect of tazobactam, ostensibly through saturation of the inhibitor, allowing the excess enzyme to hydrolyse and degrade piperacillin [24]. β-lactamase hyperproduction can occur via increased gene expression modulated by a stronger promoter [25], or an increase in gene copy number mediated by insertion sequences [26, 27] or plasmids [23]. Other mechanisms have also been identified, including production of OXA-1 [24], inhibitor-resistant enzymes such as *bla*_TEM-33 (11)_, and a single nucleotide polymorphism (SNP) at position S133G in *bla*_CTX-M-15_ found *in vitro* via random mutagenesis/error prone PCR but not yet found in clinical isolates [28].

Routine blood-culture surveillance identified the occurrence of this phenotype in *E. coli* at the Royal Liverpool University Hospital (RLUH), Liverpool, UK, between 2014 and 2017. We sought to identify the diversity of *E. coli* strains and distribution of known mechanisms of TZP in our study isolates. We compared our collection to the findings of a UK-wide collection of invasive *E. coli* isolates (*n*=1509) with publicly available phenotypic and genotypic data. This data set included the TZP-R/3GC-S phenotype as well as a piperacillin/tazobactam and third-generation cephalosporin-susceptible (TZP-S/3GC-S) phenotype

## Methods

### Study setting

The RLUH is a city centre located hospital in Liverpool, UK, providing secondary and tertiary care, with a catchment area of >2 million people in Merseyside, Cheshire, North Wales, and the Isle of Man. In 2019 the hospital recorded over 587 000 outpatient appointments and 95 000 daily inpatients.

### Surveillance data at RLUH and identification of TZP-R/3GC-S isolates

Bloodstream bacterial pathogens were isolated using the BacTAlert 3D blood-culture system (bioMérieux, France) and identified to a species level using MALDI-TOF (Bruker, USA). Antimicrobial susceptibility testing (AST) was carried out using disc-diffusion-based testing according to the British Society of Antimicrobial Chemotherapy guidelines [29] between 1st January 2014 and 7 August 2017, after which these were replaced by the European Committee for Antimicrobial Susceptibility Testing (EUCAST) guidelines [30]. In 2014 ceftazidime was used as the indicator 3GC, which was changed to cefpodoxime between 2015 and 2017. Isolate details and AST results were recorded in the Laboratory Information System (Telepath, CSC, USA). All isolates were retained in glycerol stocks at −80 °C in the RLUH Biobank. Data for the study was extracted into a database, including susceptibility data for ampicillin, cefpodoxime/ceftazidime, TZP, meropenem, ertapenem, cefoxitin, ciprofloxacin, gentamycin, amikacin, amoxicillin/clavulanic acid, tigecycline and cefalexin. The data was used to estimate the proportion of *E. coli* isolates per year with TZP resistance, with and without associated 3GC resistance. Intermediate TZP resistance was determined according to the EUCAST guidelines during the study period, with isolates with zone diameters of 20–21 mm during disc-diffusion testing falling into that category. Susceptibility or resistance was only recorded in the case of a test result; isolates without a result to a particular antibiotic were not included in the proportional analysis of resistance to that antibiotic. In cases where multiple isolates were obtained from a single infectious episode, only the first isolate was included for further investigation and sequencing, to avoid duplication. Isolates that were TZP-R/3GC-S were retrieved from the Biobank and resurrected from glycerol stocks using Luria–Bertani agar (Oxoid, UK) and incubated at 37 °C for 18 h.

### Confirmation of antimicrobial susceptibility

MIC for the isolates were obtained using the E-TEST method (Biomerieux, France) [31] according to EUCAST guidelines [30]. MICs were determined for TZP and the 3GC ceftriaxone (CTX). Isolates were not phenotypically screened for ampC β-lactamase.

### DNA extraction and sequencing

Isolates were subcultured onto Luria–Bertani agar and incubated at 37 °C. Genomic DNA was extracted from single colonies using the PureGene Yeast/Bacteria Kit (Qiagen, Germany), following the manufacturer’s instructions for Gram-negative bacteria. Genome sequencing of 65 isolates was performed by MicrobesNG (http://www.microbesng.uk), using 2×250 bp short-read sequencing on the Illumina MiSeq (lllumina, US) (Table S1).

### Genome analysis, sequence typing and AMR gene prediction

All genomes were *de novo* assembled and annotated using SPAdes version 3.7 [32], and Prokka 1.11 [33], respectively, by MicrobesNG. Trimmed and quality-filtered sequencing reads were also provided, using the in-house methodology (adapter trimming with Trimmomatic v0.30, with a sliding window quality score cutoff value of Q15, and quality assessed using FastQC 0.11). The presence and copy number of AMR genes was determined using ARIBA [34], with the SRST2 database [35]. *In silico* multi locus sequence typing (MLST), and plasmid replicon typing were carried out using ARIBA and the MLSTFinder [36] and PlasmidFinder [37] databases, respectively. β-lactamase promoters were identified by constructing databases with promoter sequences for *bla*_TEM-1_ [25] and screening the genomes for these sequences using ARIBA . Copy numbers were estimated by dividing the sequencing coverage of β-lactamase genes by the coverage of the chromosomal single-copy gene *ampH*.

### Phylogenetic analysis of study isolates

A pan-genome analysis of all sequences was generated using Roary [38], and the core gene alignment was used as input for snp-sites [39] to extract ACGT-only SNPs (-c option). A maximum-likelihood tree was produced using iqtree [40], with the general time reversible (GTR) model and gamma correction using ASC ascertainment bias correction (ASC) for SNPs-only alignments (-m GTR +G+ ASC) and 1000 bootstrap replicates (-bb 1000). Phylogenetic trees were annotated using the Interactive Tree of Life [41] (https://itol.embl.de/). Core-genome trees for sequence types ST131 and ST73 were generated by mapping the sequencing reads from each study isolate in the STs against the reference chromosomes of *E. coli* strains EC958 (HG941718.1) and CFT073 (AE014075.1), respectively, using snippy (https://github.com/tseemann/snippy). Recombination blocks were removed with Gubbins [42], and extraction of SNPs-only of the recombination-free alignment, and tree calculation, were performed as described above, using SNP-sites and IQ-TREE.

To investigate the relation of the study isolates to the whole UK hospital *E. coli* population, the sequences from a large UK-wide comparative analysis were included (PRJEB4681 [43]). These sequences included 1094 isolates submitted to the UK-wide Bacteraemia Resistance Surveillance Programme (www.bsacsurv.org) between 2001–2011 by 11 hospitals across England, and 415 isolates provided by the Cambridge University Hospitals NHS Foundation Trust, Cambridge.

A core-gene alignment and phylogenetic tree were constructed. Isolates from the UK-wide collection with the same phenotype of TZP resistance/3GC susceptibility (defined as susceptibility to both ceftazidime and cefotaxime, or either compound if only one was tested) were identified from the phenotypic AMR data [43], and highlighted alongside study isolates.

### Data availability

Raw read data and assemblies were submitted under BioProject ID PRJNA644114. Detailed per-strain information on accession numbers, resistance profiles, resistance gene predictions and STs are given in Table S2.

## Results

### Isolate collection and antimicrobial susceptibility testing

**Fig. 1.**
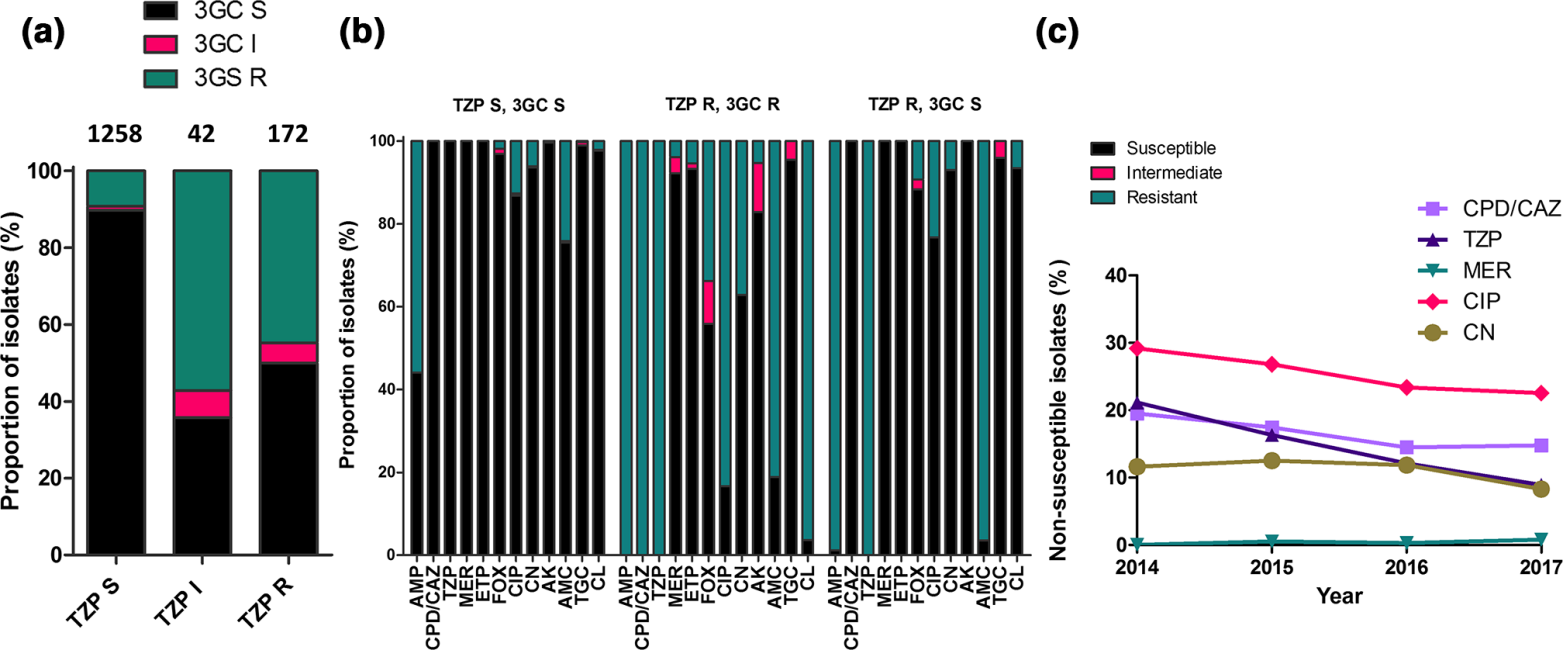
(a) Proportion of TZP susceptible (TZP S), intermediate (TZP I) and resistant (TZP R) isolates that are third-generation cephalosporin susceptible (3GC S), intermediate (3GC I) and resistant (3GC R). (b) Antimicrobial susceptibilities of *E. coli* isolates from the RLUH, grouped by their susceptibility to piperacillin/tazobactam (TZP) and third-generation cephalosporins (3GC). Susceptibility data is shown for isolates that are TZP susceptible and 3GC susceptible (TZP S, 3GC R). TZP resistant and 3GC resistant (TZP R, 3GC R), and TZP resistant and 3GC susceptible (TZP R, 3GC S), for the antibiotics ampicillin (AMP), cefpodoxime/ceftazidime (CPD/CAZ), piperacillin/tazobactam (TZP), meropenem (MER), ertapenem (ETP), cefoxitin (FOX), ciprofloxacin (CIP), gentamycin (CN), amikacin (AK), amoxicillin/clavulanic acid (AMC), tigecycline (TGC), and cefalexin (CL). (c) Trends in non-susceptibility to CPD/CAZ, TZP, MER, CIP and CN between 2014 and 2017 at RLUH.

The RLUH recorded 1472 BSI *E. coli* isolates between 2014 and 2017 and antimicrobial susceptibility testing showed 172 isolates (11.8 %) were resistant to TZP (Fig. S1). The proportion of *E. coli* resistant to TZP declined between 2014 (21 %) and 2017 (9 %, Fig. 1c). Of the 1258 TZP-susceptible isolates, the majority (1129, 89.7 %) were susceptible to 3GC, while 129 (10.3 %) were 3GC non-susceptible. In contrast, 86/172 (50 %) TZP-resistant isolates were non-susceptible and 86/172 (50 %) were susceptible to 3GC in our setting (Fig. 1a).

Resistance to carbapenems was only seen in the TZP-resistant/3GC-resistant isolates, with 3.9 % resistant to meropenem and 5.3 % to ertapenem. A higher proportion of the TZP-R/3GC-S isolates were resistant to amoxicillin/clavulanic acid in comparison with TZP-resistant/3GC-resistant isolates (96.4 vs 81.1 %) (Fig. 1b). Overall, aside from the penicillin class antibiotics, a high proportion of the TZP-R/3GC-S phenotype were susceptible to the antimicrobials tested.

Of the 86 isolates with the TZP-R/3GC-S phenotype, 14 had been derived from repeated sampling of long-term patients and were excluded, resulting in 72 isolates derived from unique patients. These isolates were reduced to 66 after excluding TZP MICs under the EUCAST breakpoint for susceptibility. A further isolate was considered a contaminant (* Staphylococcus aureus *) based on colony morphology, which was confirmed by 16S PCR. After whole-genome sequencing, two of the 65 isolates were removed as they contained more than one *E. coli* genome, either due to mixed infections or contamination (assembly sizes were 9 602 556 bp and 9 552 068 bp, respectively), leaving 63 isolates for further analysis.

The MICs of TZP as assessed by the E-TEST ranged from 12 to 256 mg l^−1^. All 63 isolates had MICs over the EUCAST breakpoint for resistance (8 mg l^−1^), with five within the EUCAST area of technical uncertainty (ATU) >8 mg l^−1^ to 16 mg l^−1^ (previously to 2020 these MICs were scored as intermediate). As these isolates fell within the ATU they were discontinued from further analysis. The CTX MICs ranged between 0.016 and 0.25 mg l^−1^, all below the breakpoint for resistance (2 mg l^−1^), confirming the TZP-R/3GC-S phenotype.

### Resistance and plasmid profile of TZP-resistant/3GC-susceptible population

The AMR genotypes (Fig. S2) correlated well with the phenotypic data obtained by disc testing, with most isolates susceptible to ciprofloxacin and gentamicin. The 58 TZP-R/3GC-S isolates harboured a variety of β-lactamase genes, including TEM-type (*n*=44; *bla*_TEM-1_ [41], *bla*_TEM-33_ [2], *bla*_TEM-148_ [1], *bla*_SHV-1_ [*n*=9], *bla*_CTX-M-15_ [*n*=4] and *bla*_OXA-1_ [*n*=3]).The presence of β-lactamase genes correlated with resistance to ampicillin and TZP, whilst resistance to ciprofloxacin in 17/58 isolates (29 %) was accounted for by *gyrA* mutations D87N (10/17, 59 %) and S83L (12/17, 71 %), and *parC* S80I mutation (10/17, 59 %). Aminoglycoside resistance was explained by the *O*-adenylyltransferases *aadA* (6/6, 100 %), in combination with the genes *aac* [3]-*IIa* or *aadB* (3/6, 50 %). Additionally, all isolates carried the chromosomal *bla*_AmpC1_, which is constitutively expressed at a low level [44], and 51/58 of the isolates carried *bla*_AmpC2_. A single isolate (169961) had a coding mutation in a penicillin-binding protein, with an A37T mutation in *mrdA* encoding penicillin-binding protein 2, in combination with the inhibitor-resistant *bla*_TEM-33_ and strong *Pa/Pb* promoter.

**Fig. 2.**
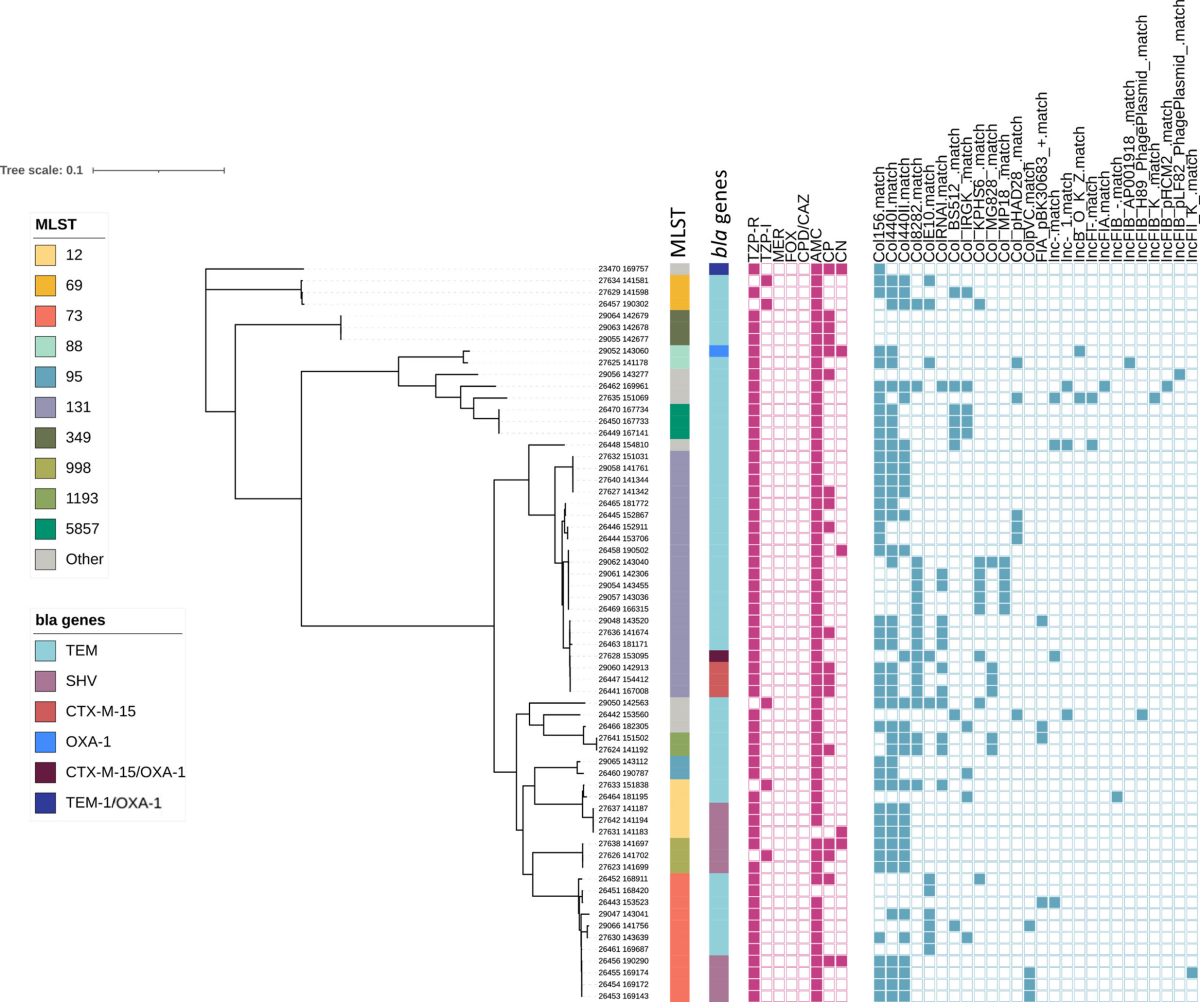
Maximum likelihood phylogeny of the study isolates from RLUH. The colour strips, from left to right, show the MLST classification (MLST), β-lactamase gene carriage (*bla* genes). The heat maps show phenotypic resistance to piperacillin/tazobactam (TZP), meropenem (MER), cefoxitin (FOX), cefpodoxime/ceftazidime (CPD/CAZ), ampicillin (AMP), ciprofloxacin (CP), and gentamycin (CN), and the plasmid replicon repertoire.

Replicons usually associated with large resistance plasmids, such as IncFIA and IncFIB, IncFIA and IncFIIA, were detected in 19 % of the study isolates (Fig. 2), reflecting the low proportion of isolates with multiple resistance genes and the unusual resistance profile characteristic of the TZP-R/3GC-S phenotype.

### Population structure of the TZP-resistant/3GC-susceptible population within the nationwide context

**Fig. 3.**
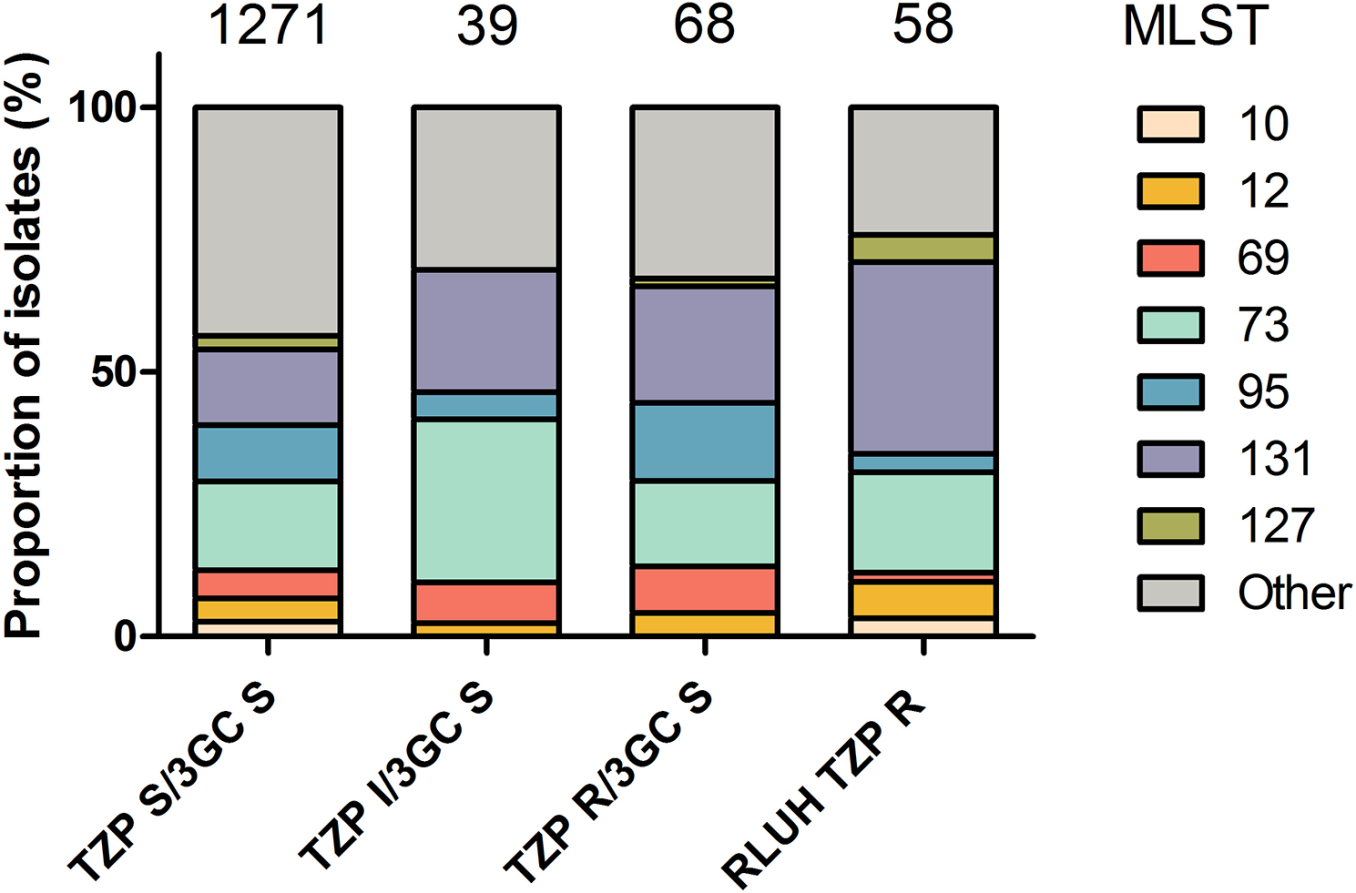
Bar chart showing the proportion of isolates belonging to common sequence types in the RLUH study isolates in comparison with those in the collection of 1509 isolates taken from a UK-wide study [43].

**Fig. 4.**
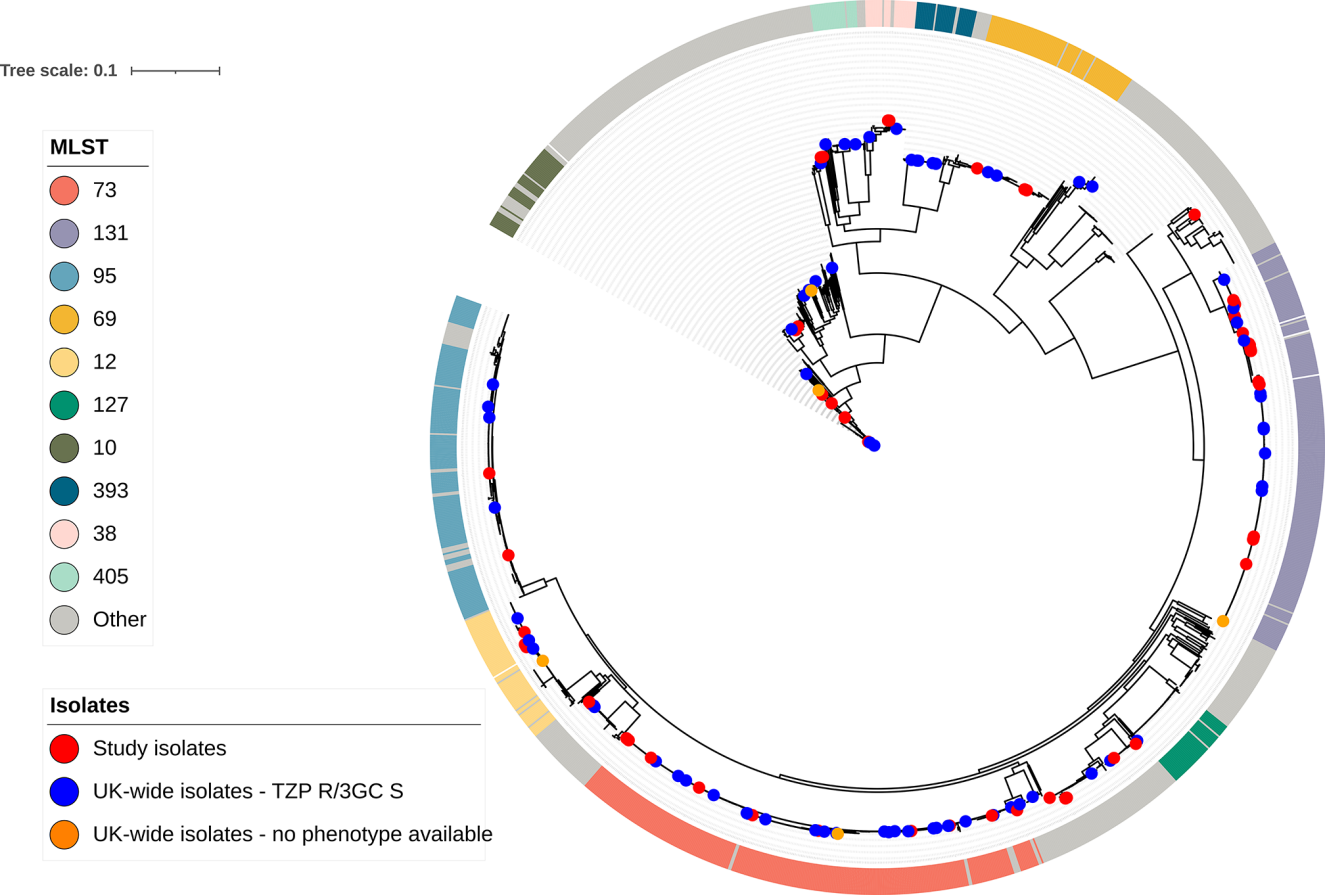
Circular maximum-likelihood core-genome phylogenetic tree of the 68 study isolates in combination with 1509 UK-wide study isolates. The ring indicates the ten most commonly encountered STs. Dots at the terminus of branches indicate study isolates, UK-wide isolates with the TZP resistant/3GC susceptible phenotype (TZP-R/3GC -S) or isolates from the UK-wide collection missing sufficient phenotypic data to assign an accurate AMR phenotype.

Phylogenetic analysis revealed the TZP-R/3GC-S phenotype occurred in a diverse number of sequence types (Fig. 2). The 58 TZP-R/3GC-S isolates represented 16 STs. The most representative were ST131 (36.2 %), ST73 (19 %) and ST12 (6.9 %). The TZP-R/3GC-S phenotype in the UK-wide collection was similarly diverse to the RLUH collection with ST131 (22.1 %) the most represented, followed by ST73 (16.2 %) and ST95 (14.7 %, Fig. 3). This diversity was also reflected in the TZP-S/3GC-S phenotype; ST73 (16.8 %), ST131 (14.3 %) and ST95 (10.6 %). When placing the RLUH isolates into the phylogenetic context of the UK-wide bloodstream isolates collected from 2001 to 2011, it was apparent that they reflected the overall *E. coli* population structure (Fig. 4). This indicates that the TZP-R/3GC-S phenotype is not driven by a clonal outbreak within this single hospital setting, but rather by multiple acquisitions of resistance mechanisms in the circulating population of hospital strains. The AMR gene profile of the RLUH isolates varied between STs (Fig. S3), with ST131 carrying more AMR genes than the other major STs, as previously reported [45]. To get a higher-resolution insight into the within-ST diversity of the isolates, we calculated core-genome trees of the main STs by mapping the reads against selected reference genomes and extracting the conserved, non-recombinant SNPs (Figs S4 and S5). The acquisition of the phenotype was not a single event even in these closely related organisms, as it occurred on several occasions for both main sequence types, with no (ST73) or very few (ST131) isolates closely related, which may indicate within-hospital transmission.

### Varied putative genetic determinants of the TZP-resistant/3GC-susceptible phenotype

**Fig. 5.**
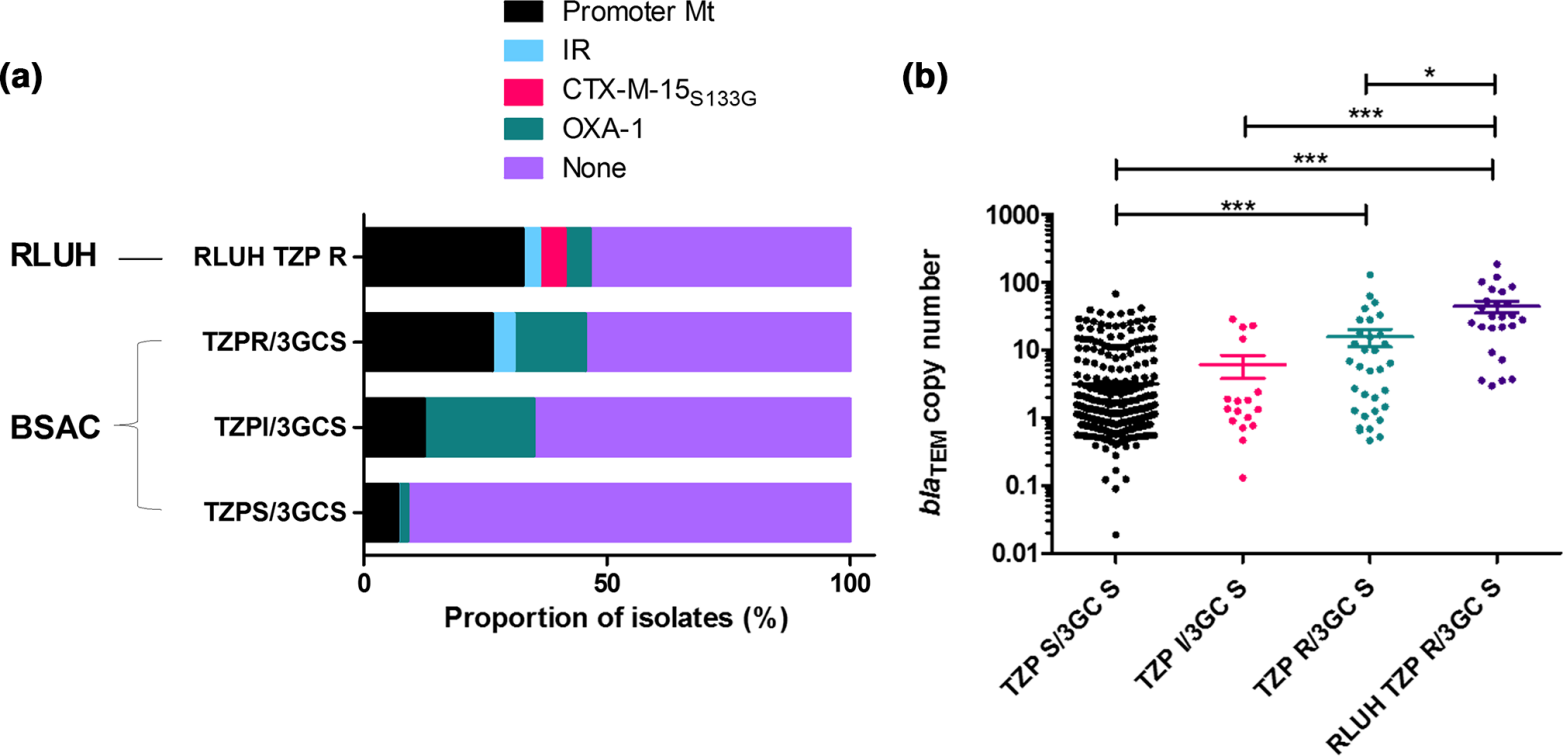
Proportion of isolates of each phenotype identified in the RLUH and BSAC collections with identifiable putative TZP resistance mechanisms (IR; inhibitor resistance) (a), and the copy number of *bla*_TEM-1_ genes found in isolates belonging to each phenotype (b), with significance determined by Dunn’s multiple comparison test.

We sought to identify previously published putative resistance mechanisms associated with the TZP-R/3GC-S phenotype in the 58 isolates from RLUH (Fig. 5a). No carbapenemase genes were predicted to be present, although four ST131 isolates harboured the ESBL *bla*_CTX-M-15_ gene, normally associated with 3GC resistance. However, three isolates carried a SNP resulting in the non-synonymous amino acid change from serine to glycine at position 133. This amino acid change is reported to result in a non-ESBL phenotype with increased TZP resistance. However the S133G mutation was only identified through random mutagenesis/error prone PCR *in vitro* [28]. In the remaining isolate with *bla*_CTX-M-15_ the promoter sequence was deleted and therefore presumably not expressed (Fig. S6). However, the isolate also carried *bla*_OXA-1_. In all three isolates carrying *bla*_OXA-1_, it was either the sole β-lactamase or it was carried with a second β-lactamase. Of the isolates with *bla*_TEM-1_, 25 had the weak *P3* promoter, four had the strong promoter *P4* and 12 contained the strong, overlapping promoter *Pa/Pb*. The *P4* and *Pa/Pb* promoter have previously been linked to hyperproduction of TEM-1 [25]. The TZP-R/3GC-S phenotype has previously been associated with increases in the copy number of *bla*_TEM-1_ via gene amplification, resulting in hyperproduction of the TEM-1 enzyme [22, 26]. The copy numbers of *bla*_TEM-1_, as estimated by sequencing coverage, for those isolates within the RLUH collection with a weak *P3* promoter varied between 3 and 186 copies, and a mean of 44 copies (Fig. 5b).

We identified inhibitor resistant β-lactamases (3; 4%), *bla*_OXA-1_ (10, 14.7%) and *bla*_TEM-1_ promoter region mutations (18; 26%) in the 68 TZP-R/3GC-S isolates from the UK-wide collection (Fig. 5a). However, we also identified these mechanisms, although at a lower incidence (inhibitor resistant β-lactamase; 2 [0.2 %], *bla*_OXA-1_ 24 [1.8 %] promoter region mutations; 89 [7 %]), in the TZP-S/3GC-S phenotype in the same collection. In total a putative mechanism was found in 27 of the 58 isolates.

The copy number of *bla*_TEM-1_ was also elevated in both the TZP-R/3GC-S (min-max of 0.5 and 129 copies, mean 16 copies) and TZP-S/3GC-S (min-max of 0.02 and 68 copies, mean three copies) from the UK-wide collection (Fig. 5b). Despite this, there was a significant difference in copy number between the TZP-S/3GC-S vs UK-wide TZP-R/3GC-S phenotypes (*P* value <0.001; Dunn’s multiple comparison test) and the TZP-S/3GC-S vs TZP-R/3GC-S phenotypes from RLUH (*P* value <0.001; Dunn’s multiple comparison Test). This indicates that although an increase in copy number of *bla*_TEM-1_ may not always be predictive of TZP-R/3GC-S, it is associated with the phenotype.

## Discussion

This phylogenetic analysis of the TZP-R/3GC-S phenotype in *E. coli* from RLUH demonstrates that this phenotype derives from repeated, multiple acquisition events. Our comparison of the RLUH isolates with a large UK-wide collection [43] shows that this is not unique to our study site, but broadly reflective of the phenotype from multiple sites across the UK. As the TZP-R/3GC-S phenotype also reflects the overall UK population structure of *E. coli* bacteraemia isolates, this is suggestive of the impact of repeated or sustained antimicrobial pressure, rather than fixation in a certain lineage and subsequent spread. The phenotype was encountered in the typically drug-resistant ST131 [46], and the often highly virulent but drug-susceptible ST73 [47], reflecting the overall dominance of these STs, and was not associated with an overall increase in carriage of genes conferring resistance to other classes of antibiotics. We also were unable to identify the presence of a common plasmid replicon, further highlighting the diversity of the phenotype. The 3GC-S phenotype was common amongst TZP-R isolates at our setting, and also occurred in 81 % of TZP-R isolates in the larger UK-wide collection.

Strategies to increase the effectiveness of TZP include increasing dosage, which in one study increased the coverage of TZP from 83.2–93 % of bacterial bloodstream pathogens [48]. Increasing the concentration of tazobactam alongside a fixed dose of piperacillin has also rescued TZP effectiveness against TEM-1 hyperproducers in a neutropenic mouse model [24], and could be a viable strategy to protect its future effectiveness. It is worth noting observational clinical data [49], and *in vivo* experimental data [50], suggesting TZP may be effective against some organisms with *in vitro* phenotypic resistance to TZP. In 2020 EUCAST updated their guidelines to reclassify isolates with TZP MICs between 8 and 16 mg l^−1^ from intermediate to ‘Susceptible, increased exposure’ (I). The aim of this change was to indicate that in some infection sites it may be possible to successfully use an increased dose for isolates with MICs within this range.

The rapid identification of the TZP-R/3GC-S phenotype could potentially enable de-escalation from TZP to a 3GC [51], both reducing the likelihood of treatment failure, and preventing overuse of carbapenems, which is key for antimicrobial stewardship [52]. However, care must be taken in this approach to reduce the risk of treatment failure, as it is unknown whether the mechanisms of TZP-R may readily evolve to confer resistance to 3GCs due to selection pressure during antimicrobial therapy. The isolates were also mostly susceptible to ciprofloxacin, gentamicin and amikacin, providing further de-escalation opportunities. There was also a major difference in cefalexin resistance, with 6.5 % of the TZP-R/3GC-S isolates resistant to cefalexin, in comparison with 96.3 % of the TZP-R/3GC-R isolates. Molecular diagnostics and whole-genome sequencing can be used to rapidly detect AMR genes to predict AMR phenotype [53, 54]. However, it is essential to match the phenotypic resistance to the genotypic mechanisms. The majority of TZP-R/3GC-S isolates in this study hyperproduced the class A β-lactamase enzymes *bla*_TEM-1_, which can hydrolyse piperacillin but not 3GCs, and is inhibited by tazobactam. Hyperproduction can occur via gene amplification, in which tandem repeats of AMR genes are generated, for example via the IS*26*-mediated amplification of pseudo-compound transposons [26, 55], or the transfer of β-lactamase genes to high copy AMR plasmids [23]. A number of the isolates were lacking a detectable increase in gene copy number, but had a potential route to hyperproduction via a strong promoter of *bla*_TEM-1_ [25]. Hyperproduction is thought to overcome the inhibitory effect of tazobactam, by saturating the inhibitor and allowing for the hydrolysis of the antibiotic. It is unclear what level of copy number or hyperproduction is required to cause resistance, and this likely differs between *E. coli* strains, dependent on genetic background. Further studies are required to better understand the effect of *bla* gene copy number on resistance to β-lactam/inhibitor combinations, especially for predicting resistance from sequencing data.

We also detected *bla*_TEM-33_, encoding an inhibitor resistant variant of TEM-1B [56], and *bla*_OXA-1_, either as the only β-lactamase or in combination with *bla*_TEM-1_. OXA-1 is poorly inhibited by tazobactam [57] but has been associated with the TZP-R/3GC-S phenotype [58], while a recent UK study identified *bla*_OXA-1_ as a major contributor to TZP resistance amongst ESBL *E. coli* [58]. However, the carriage of *bla*_OXA-1_ does not always confer resistance to TZP, which appears to depend on the genetic background of the strain. The risk ratio of *bla*_OXA-1_ being associated with TZP resistance in ESBL *E. coli* is higher in ST131 strains (12.1) compared with ESBL *E. coli* as a whole (6.49) [58]. One isolate carrying the OXA-1 β-lactamase gene, as well as *bla*_CTX-M-15_ lacking a promoter, belonged to ST131.

Three out of four detected *bla*_CTX-M-15_ encoded the S133G mutation, which increases TZP MIC ten-fold, whilst reducing the 3GC MIC by the same margin in a strain harbouring a random mutagenesis/error prone PCR derived *bla*_CTX-M-15_ [28]. To our knowledge this is the first report of this *bla*_CTX-M-15_ variant in clinical isolates. The S133G mutation in *bla*_CTX-M-15_ was associated with 5 % of TZP-R/3GC-S in our setting and only in ST131. The mutation of *bla*_CTX-M_ genes to better hydrolyse mecillinam has been reported during urinary tract infection treatment [59], but not for TZP or other β-lactam/inhibitor combinations. The circulation of *bla*_CTX-M_ variants that do not confer the ESBL phenotype but provide resistance to TZP, has implications for molecular testing for ESBL organisms [60], as it would misclassify the isolates as 3GC-resistant and lead to unnecessary use of carbapenems. We found that 9 of 58 isolates, all without a putative resistant mechanism, harboured blaSHV-48. Hyperproduction of this enzyme has been shown to lead to the TZP-R/3GC-S phenotype in *K. pneumoniae* [61].

All the putative mechanisms of TZP-R/3GC-S found in the isolates from RLUH have been previously published and widely associated with this phenotype. However, also we found evidence of *bla*_TEM-1_ promoter region mutations, inhibitor resistance enzymes and increased *bla*_TEM-1_ copy number in the TZP-S/3GC-S phenotype. The only putative mechanism, which was not found in the TZP-S/3GC-S phenotype, was the S133G mutation in *bla*_CTX-M-15_. This mutation was not found in any of the TZP-R/3GC-S phenotype isolates from the UK-wide collection, which may indicate low incidence or a localized emergence in our hospital. The diverse putative mechanisms of TZP-R/3GC-S and phenotype-genotype discordance, as seen in TZP-S/3GC-S, would compromise current molecular or genomic detection of this phenotype. Further studies are required to experimentally validate the effect of these putative mechanisms on TZP resistance to improve our understanding of this phenotype, particularly in clinical isolates.

The main limitation of this study was that only TZP-R/3GC-S isolates from the RLUH were sequenced, and the relatively small population size. We utilized a large and UK-wide collection of isolates for comparison, which were similarly diverse and reflected the overall population structure [43]. Additionally, we relied on the routine AST carried out in the hospital to classify the isolates and did not carry out confirmatory MIC determination using ceftriaxone/cefpodoxime. In 2014 ceftazidime was the indicator cephalosporin used, which is insensitive for identifying CTX-M producers. This may have caused us to overestimate 3GC-S isolates during this year, however only a single TZP-R/3GC-S isolate from this year was found to have a *bla*_CTX-M_, which carried the S133G mutation, showing we did not erroneously include CTX-M producers likely to be 3GC-R. The lack of inclusion of patient-level treatment data means that we cannot link infections with *E. coli* isolates displaying the TZP-R/3GC-S phenotype to a particular treatment regimen. For our description of the hospital surveillance data, we used the routine AST at RLUH to determine the portion of isolates susceptible or non-susceptible to TZP, 3GC and other antibiotics. Subsequent assessment of isolates displaying the TZP-R/3GC-S phenotype found that a subset had to be discounted from further analysis due to factors including contamination, TZP susceptibility and mixed samples. We did not retroactively remove these isolates from the analysis as we could not be certain when contamination, mixing of samples or loss of resistance occurred.

This work highlights the phylogenetic diversity of the TZP-R/3GC-S phenotype in *E. coli* and the variety of the putative resistance mechanisms involved, including β-lactamase hyperproduction via gene amplification and promoter mutations, inhibitor-resistant TEM-1 and CTX-M-15 variants. However, the presence of these mechanisms at a lower incidence with the TZP-S isolates highlights that a greater understanding of the evolution of TZP resistance and the resistance mechanisms of the TZP-R/3GC-S phenotype would be fundamental to improve the prediction of TZP-R/3GC-S *E. coli *. Until such time, phenotypic monitoring of this phenotype is essential to prevent treatment failure.

## Supplementary Data

Supplementary material 1

Supplementary material 2
