# Supplementary material for: Piperacillin/tazobactam-resistant, cephalosporin-susceptible Escherichia coli bloodstream infections are driven by multiple acquisition of resistance across diverse sequence types
Source: Microb Genom. 2022 Apr 11;8(4):000789. doi: 10.1099/mgen.0.000789 (PMC9453079; doi:10.1099/mgen.0.000789)
Supplement: Supplementary material 1 [file mgen-8-0789-s001.pdf]

Supplementary Figures

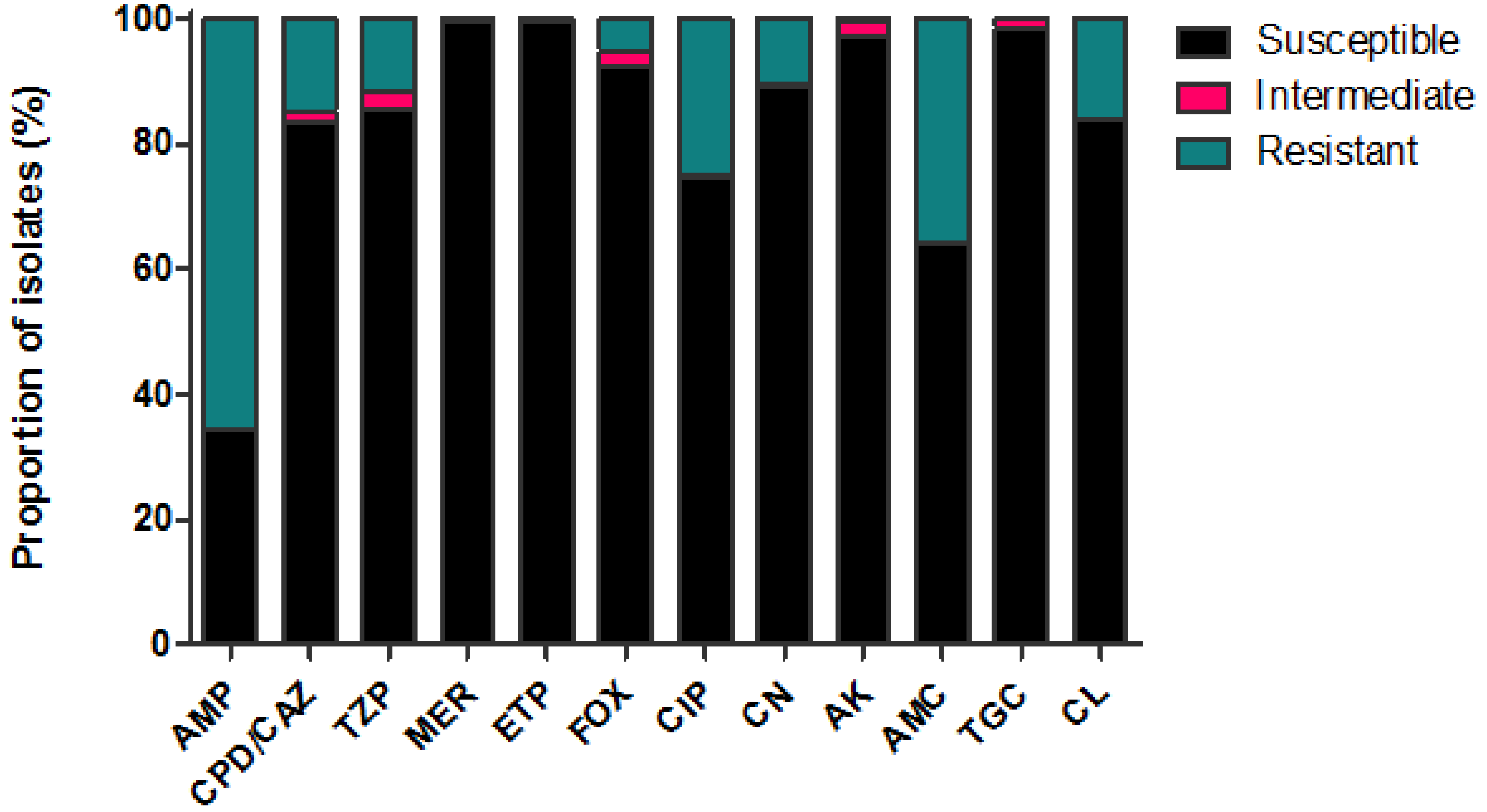

Fig.S1. Proportion of the total *E. coli* isolated from blood stream infections between 2014 and 2017 at RLUH that were susceptible, intermediate or resistant to ampicillin (AMP), cefpodoxime/ceftazidime (CPD/CAZ), piperacillin/tazobactam (TZP), meropenem (MER), ertapenem (ETP), cefoxitin (FOX), ciprofloxacin (CIP), gentamycin (CN), amikacin (AK), amoxicillin/clavulanic acid (AMC), tigecycline (TGC), and cefalexin (CL)

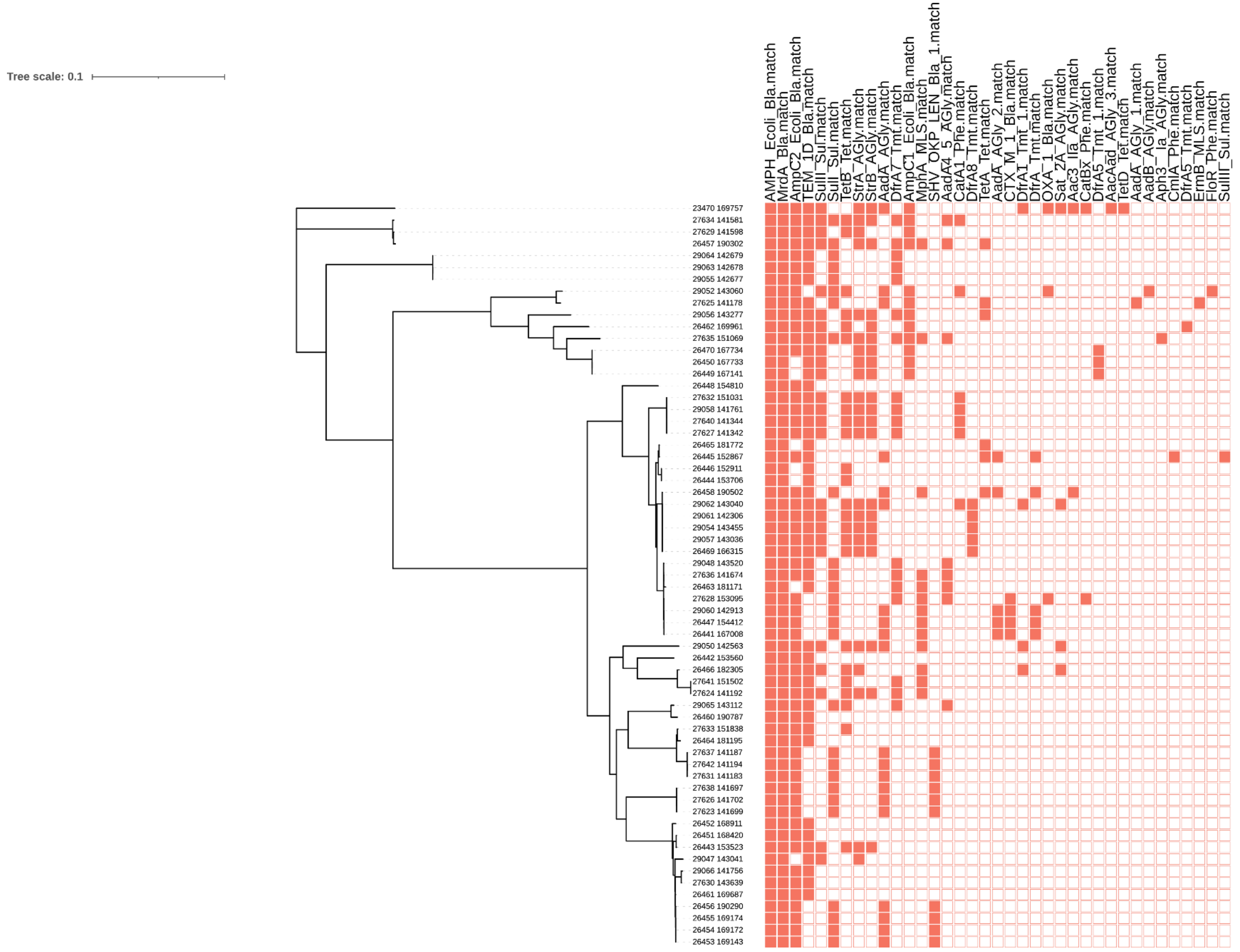

Fig.S2 Maximum likelihood phylogeny of the study isolates from RLUH, with a heat map indicating the AMR gene repertoire.

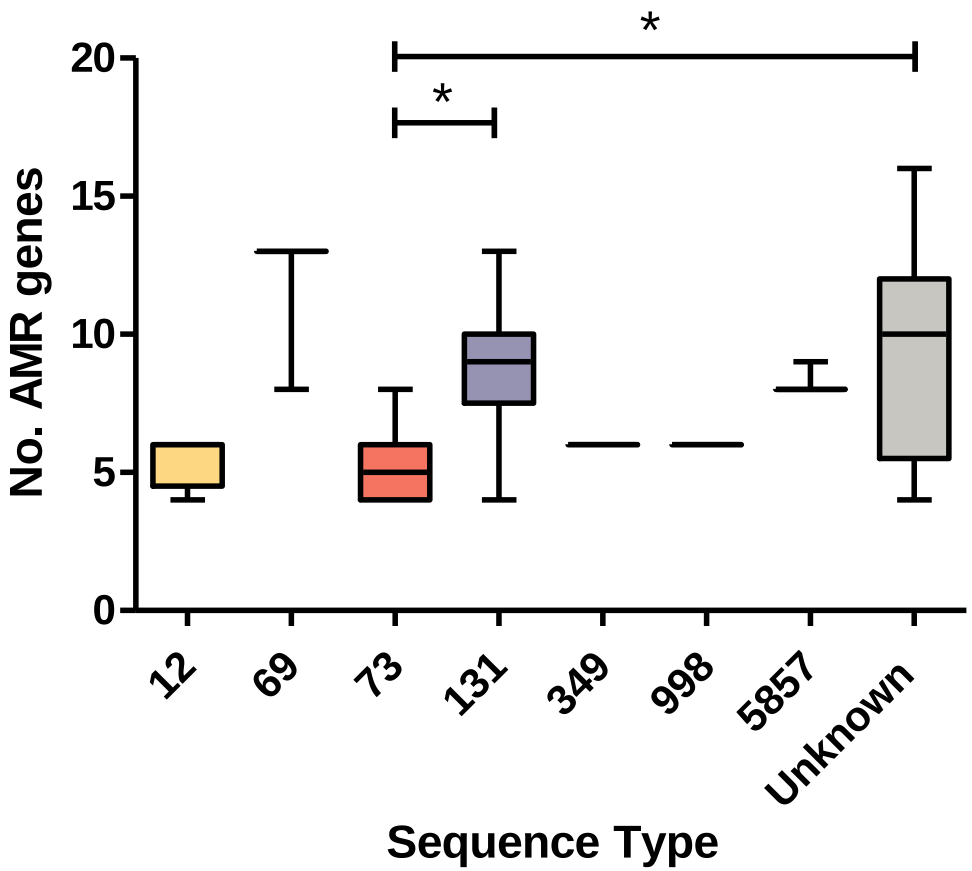

Fig.S3 The number of AMR genes in isolates from the major sequence types encountered in the study. Whiskers show minimum and maximum values. Significance determined by Kruskal-Wallis test, * indicates a p value of <0.05.

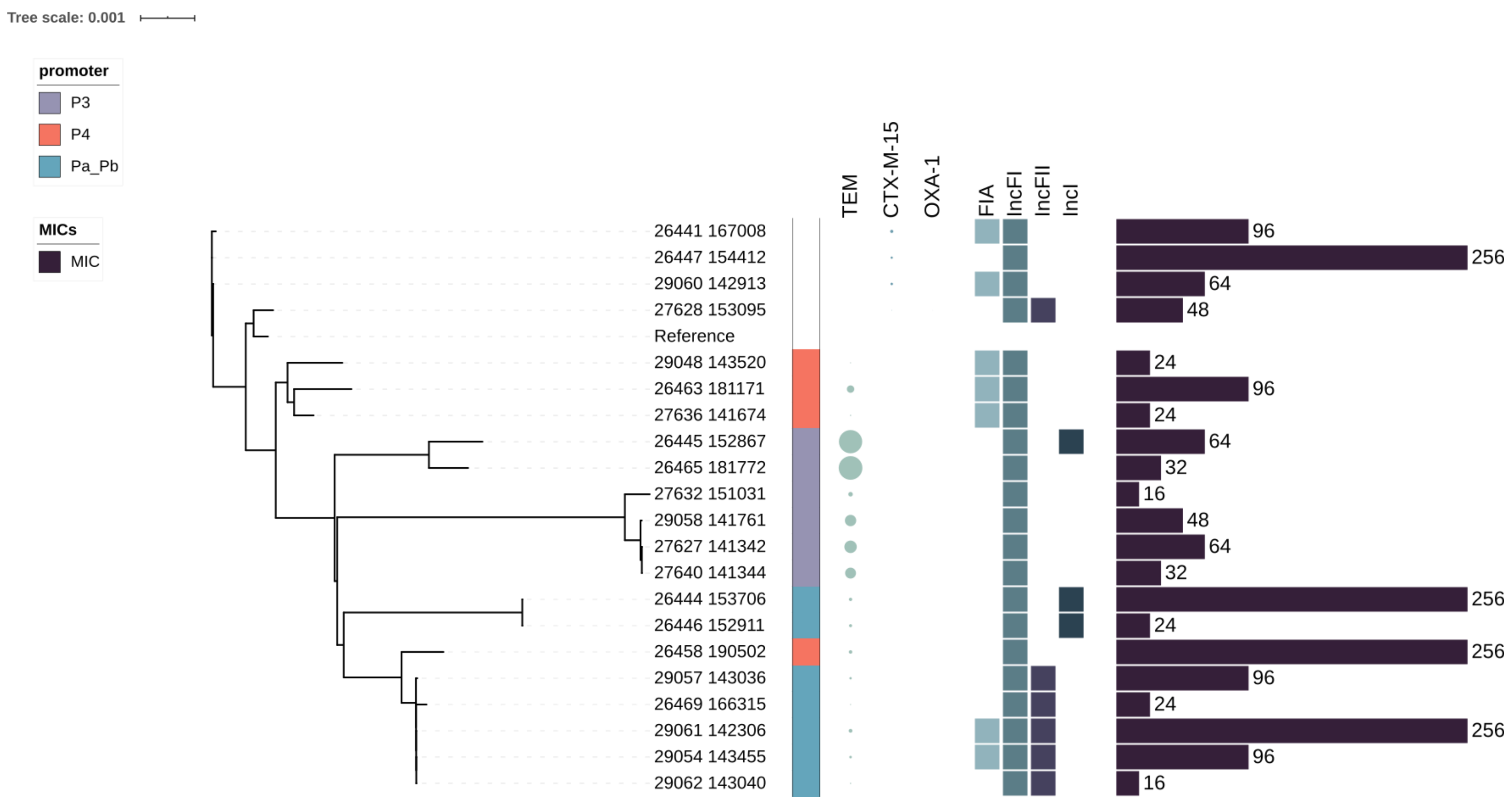

Fig.S4. High resolution core genome – based phylogeny of TZP resistant/3GC susceptible ST131 isolates. Indicated are promoter types, β-lactamase copy numbers (size of circle represents relative copy number), plasmid replicons, and TZP MIC.

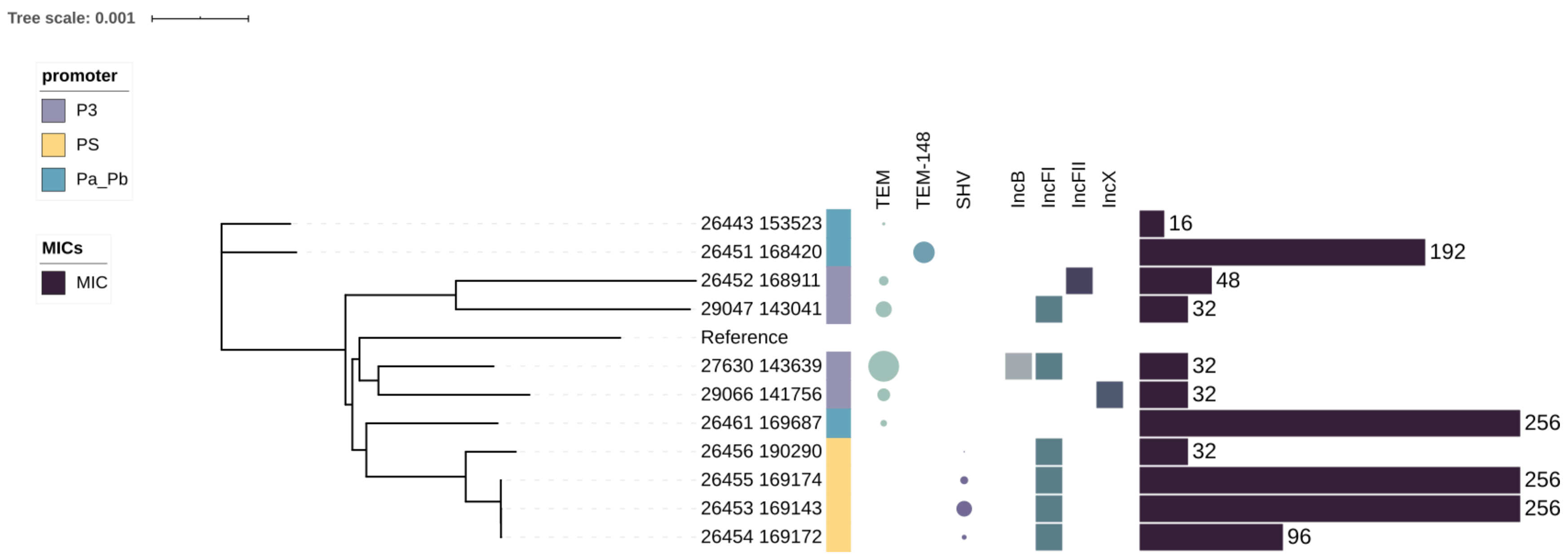

Fig.S5. High resolution core genome – based phylogeny of TZP resistant/3GC susceptible ST73 isolates. Indicated are promoter types, β-lactamase copy numbers (size of circle represents relative copy number), plasmid replicons, and TZP MIC.

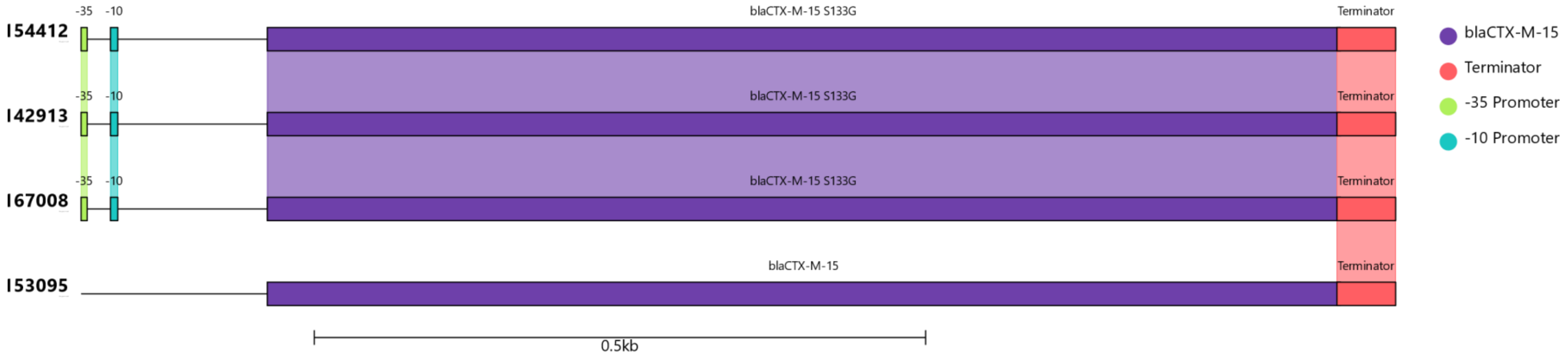

Fig.S6: Comparison of the promoter region, gene and terminator of blaCTX-M-15 predicted to be present in four clinical isolates from the Royal Liverpool University Hospital collection. Isolates 154412, 142913 and 167008 all contained an intact promoter and terminator with a blaCTX-M-15 containing the S133G mutation, while isolate 153095 harboured a wild type blaCTX-M-15 but lacking the promoter. Shaded regions between isolates indicate 100% identity. Figure produced using clinker.
